# Disentangling the stigma of HIV/AIDS from the stigmas of drugs use, commercial sex and commercial blood donation – a factorial survey of medical students in China

**DOI:** 10.1186/1471-2458-7-280

**Published:** 2007-10-05

**Authors:** Kit Yee Chan, Yi Yang, Kong-Lai Zhang, Daniel D Reidpath

**Affiliations:** 1 School of Health and Social Development, Deakin University, Burwood Highway, Burwood, Victoria 3125, Australia; 2 Department of Epidemiology, Guangdong College of Pharmacy, Guandong Province 510224, China; 3 Peking Union Medical College, 5 Dong Dan San Tiao, Beijing 100005, China; 4 Centre for Public Health ResearchBrunel University, Uxbridge, Middlesex, UB8 3PH, UK

## Abstract

**Background:**

HIV/AIDS related stigma interferes with the provision of appropriate care and support for people living with HIV/AIDS. Currently, programs to address the stigma approach it as if it occurs in isolation, separate from the co-stigmas related to the various modes of disease transmission including injection drug use (IDU) and commercial sex (CS). In order to develop better programs to address HIV/AIDS related stigma, the inter-relationship (or 'layering') between HIV/AIDS stigma and the co-stigmas needs to be better understood. This paper describes an experimental study for disentangling the layering of HIV/AIDS related stigmas.

**Methods:**

The study used a factorial survey design. 352 medical students from Guangzhou were presented with four random vignettes each describing a hypothetical male. The vignettes were identical except for the presence of a disease diagnosis (AIDS, leukaemia, or no disease) and a co-characteristic (IDU, CS, commercial blood donation (CBD), blood transfusion or no co-characteristic). After reading each vignette, participants completed a measure of social distance that assessed the level of stigmatising attitudes.

**Results:**

Bivariate and multivariable analyses revealed statistically significant levels of stigma associated with AIDS, IDU, CS and CBD. The layering of stigma was explored using a recently developed technique. Strong interactions between the stigmas of AIDS and the co-characteristics were also found. AIDS was significantly less stigmatising than IDU or CS. Critically, the stigma of AIDS in combination with either the stigmas of IDU or CS was significantly less than the stigma of IDU alone or CS alone.

**Conclusion:**

The findings pose several surprising challenges to conventional beliefs about HIV/AIDS related stigma and stigma interventions that have focused exclusively on the disease stigma. Contrary to the belief that having a co-stigma would add to the intensity of stigma attached to people with HIV/AIDS, the findings indicate the presence of an illness might have a moderating effect on the stigma of certain co-characteristics like IDU. The strong interdependence between the stigmas of HIV/AIDS and the co-stigmas of IDU and CS suggest that reducing the co-stigmas should be an integral part of HIV/AIDS stigma intervention within this context.

## Background

HIV/AIDS has been described as "the most devastating epidemic humanity has even known" [1]. Over 40 million people globally are estimated to be living with HIV/AIDS, with developing countries bearing a disproportionate burden of new cases[2]. Recent projections indicate that unless effective intervention strategies are implemented, HIV/AIDS incident cases will escalate rapidly, particularly in Eastern Europe and Asia [2]. Although treatment, care, and support programs for people living with HIV/AIDS (PLWHA) are recognised as central to the global management of the epidemic [2-4], HIV/AIDS-related stigma remains a recognised obstacle to the successful implementation of such programs [2,4,5]. While negative public attitudes towards PLWHA presents a difficult social issue, prejudicial attitudes by health care professionals can directly affect access to care at the point of service delivery [6,7]. In this regard, reducing HIV/AIDS stigma is an integral part of a comprehensive approach to the delivery of appropriate treatment and care [8].

To reduce HIV/AIDS stigma, one must understand the nature of the stigma, especially in the context of the low success rates reported by existing stigma intervention studies [9]. One reason for the lack of success of these interventions may lie in the multidimensional nature of HIV/AIDS stigma. Although HIV/AIDS stigma is treated as if it is a singular entity, PLWHA are also stigmatised by virtue of stigmatised behaviours associated with the routes of transmission such as injecting drug use (IDU) and commercial sex (CS) [10,11] – referred to here as co-stigmas. A PLWHA seeking treatment in a health care centre who is also an injection drug user may be stigmatised because of the HIV/AIDS, the IDU, or a combination of the two. These co-stigmas, however, are generally ignored in stigma intervention programs, even though they will both have implications for the delivery of services to PLWHA.

The presence of co-stigma has been referred to as 'double stigma', 'triple stigma', or layers of stigmas [12-14], and a number of approaches have been developed to study this phenomenon [12,15-17]. One quantitative approach measures levels of stigmatising attitudes towards people with HIV/AIDS and/or related co-stigmatised attributes, typically using a series of written descriptions (vignettes) to elicit attitudes [18-22].

Study participants, including physicians, nurses, social workers and psychologists, have been found to respond significantly more harshly towards vignettes describing PLWHA than towards vignettes describing a less value-laden disease such as leukaemia [20,23,24]. Similarly, vignettes describing people with co-stigmas such as IDU or homosexuality result in significantly harsher responses than vignettes describing people with control characteristics such as being heterosexual or having received a blood transfusion [17,22,25].

What remains unanswered in these earlier studies, however, is how the stigma of HIV/AIDS is layered with the stigma of a co-characteristic such as IDU. Understanding this is relationship is potentially critical for the development and management of health campaigns and programs targeting stigma reduction, which have thus far focussed on the stigma of HIV/AIDS in isolation from its co-stigmas [9].

Until very recently, one of the impediments to the quantitative analysis of the relationships between stigmas had been the lack of an analytic framework. An approach to the empirical analysis of HIV layering was identified, but it provided no original data to support its use [26]. Essentially, the paper showed how the total stigma of a person with two (or more) stigmatised characteristics could be partitioned. Details of the actual approach are described in that paper. In brief, however, consider a two-by-two table representing the level of stigma experienced by people who are HIV positive versus HIV negative, and/or injection drug users versus not injection drug users. Each cell in the table represents the average level of stigma experienced by those four possible combinations: people who are neither injection drug users nor HIV positive, people who are either injection drug users or HIV positive (but not both), people who are both HIV positive and injection drug users. For those individuals with only one of the two possible stigmas it is possible to write of the 'unique' stigma associated with a characteristic in the absence of the other characteristic – crudely, the cell mean. The value of the cell representing people with the combination of the characteristics represents a potentially unique HIV stigma, a potentially unique injection drug use stigma, and either an additional or a reduced stigma in virtue of having both characteristics.

**Figure 1:**
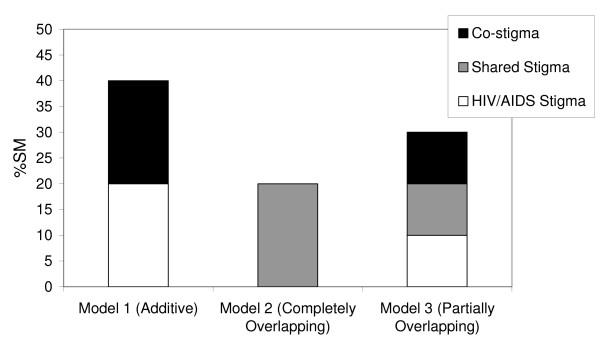
Three models of layering.

Following this analytic framework, Figure 1 illustrates three models of the inter-relationships between stigmas in an individual with two stigmatised characteristics, such as HIV/AIDS and IDU.

Model 1 illustrates the stigma of HIV/AIDS and a co-stigma assuming that they are strictly additive. In this case each source of stigma may be addressed independently and is effectively how HIV/AIDS stigma is currently treated. Model 2 illustrates the stigma of HIV/AIDS and a co-stigma assuming that the two sources of stigma completely overlap with one another. Removing the stigma of either one would have no effect on the total level of stigma. The final model portrays a compromise between the two earlier models, and is hypothesised to be the most likely portrayal of the actual relationship between the disease stigma of HIV/AIDS and its co-stigmas. It suggests that the overall stigma is composed of (i) a portion of stigma uniquely associated with being HIV-positive; (ii) a portion that is uniquely associated with the co-stigma, and (iii) a portion that is the shared effect of the two stigmas. In this case an intervention addressing only one source of stigma will only remove that relatively small portion unique to that stigma (i.e. the white portion). To address the total stigma of HIV/AIDS, one would need to deal with both the stigma of HIV/AIDS and the co-stigma.

The aim of this study is three fold. It is the first empirical test of the framework proposed by Chan and Reidpath [26]; and describes a study design and analytic approach that allows the disentangling of the inter-relationships (or layering) between the disease stigma of HIV/AIDS and its co-stigmas. It provides data from a setting from which little data has previously been obtained (Southern China), and yet has important implications for the delivery of care and support to PLWHA and the development of appropriate stigma interventions. Finally, methodological weaknesses of the framework can be assessed.

Medical students were the study population. The choice was driven in part for reasons of convenience, but largely because as future health care providers, their attitudes towards PLWHA have important ramifications for a health care system's capacity to cope with increasing demand determined by the epidemic [16,17,22].

## Methods

The study employed an adaptation of a factorial survey design. Vignettes were presented to participants and attitudinal evaluations about each vignette were recorded.

### Participants

Three hundred and sixty second year medical students at a university in Guangzhou were invited to participate. Eight elected not to. The 352 participants were almost evenly split between males and females (male = 170, female = 169, 13 unidentified) had a mean age of 20 (age range = 16–24, SD = 0.85).

### Setting

The study was conducted in Guangzhou, Southern China, a region identified as having one of the fastest growing HIV/AIDS epidemics in the world [27]. Despite a relatively low HIV/AIDS prevalence of 1933 cumulative cases, the reported HIV/AIDS incidence in Guangzhou increased by 212% in the 12 months to September 2004, from 262 to 511 cases [27].

Guangzhou is also a fertile location for this study because of China's long history of stigmatisation of behaviours associated with HIV/AIDS transmission including IDU and prostitution. Currently, 73% of all new cases of infection are associated with IDU, while 20% were associated with sexual transmission [27].

### Materials

#### Vignettes

The surveys utilised 15 possible vignettes designed to capture the interplay between a disease (no disease, AIDS, leukaemia) and a potentially stigmatised co-characteristic (no co-characteristic, blood transfusion, commercial blood donation, visited commercial sex workers, IDU). Adapted from the work of Kelly et al., each vignette was constructed in three parts. Part 1 was a general description of the 'idealised son' that was constant across all 15 vignettes, and created to conform with China's traditional hierarchical principles on the roles and duties occupied by different subjects in relation to the family and the state [28,29]:

Male 'A' was bright and had many talents. He was considered to be a dutiful son to his parents, and a kind, selfless and responsible person by everyone who knew him.

When presented on its own without further elaboration, it served as the base condition against which all others could be contrasted. In those vignettes in which Part 2 was included it described 'A"s deteriorating health:

Over the past six months, he developed a range of health problems including fatigue, physical decline and recurrent infections. He learned from his doctor that he was seriously ill, and his family and friends were said to have difficulty adjusting to his life-threatening illness.

Part 2 ended with a diagnosis of either AIDS or leukaemia:

He was diagnosed with [AIDS/leukaemia].

Part 3 of the vignette, when it was included, gave additional information about one of the following co-characteristics:

Two years ago, he received a blood transfusion as a routine part of a surgical procedure.

OR

Over the past two years, he has sold his blood on a number of occasions;

OR

For the past two years, he has occasionally visited commercial sex workers.

OR

For the past two years, he has occasionally engaged in injecting drug use for recreational purposes;

A vignette was created from each one of the possible 3 × 5 (i.e. disease by co-characteristic) combinations, giving 15 possible vignettes in total.

#### Independent variables and covariates

There were two independent variables. There was the *disease *factor with three levels (none, leukaemia, AIDS), where 'none' represented the base condition of no disease and, consistent with previous studies, leukaemia represented a control disease. There was a second factor of *co-characteristics *with five levels (none, blood transfusion, blood donation, commercial sex, IDU), where 'none' represented the base condition of no co-characteristic, and blood transfusion represented the control co-characteristic. Given the sample size, there is a restriction on the number of co-stigmatised factors that could be sensibly explored in one study. Whilst homosexuality is a common co-stigmatised factor included in previous studies, in the current study, the co-stigmatised factors were selected on the basis of their significance in terms of HIV transmission in the local epidemic.

*HIV/AIDS knowledge *was treated as a covariate because previous studies have shown that misconceptions regarding HIV transmission can lead to avoidance attitudes because of fear of infection from casual contact [30-32]. HIV/AIDS knowledge was measured using an 11-item scale of Yes/No questions designed to test participants' knowledge about the transmission of HIV/AIDS. These questions were adapted from a number of knowledge scales [15,33]. A score of 0 indicated no correct responses; and a score of 11 indicated all correct responses.

The other potential covariates included were age and gender.

#### Dependent (outcome) variable

Stigma was operationalised in terms of social distance, a widely used measure of the degree to which a person is willing to share a proximate social space with another [34-36]. The scale, adapted by Kelly and colleagues [17,20,37], was composed of eight items measuring participants' willingness to interact with the hypothetical person 'A' [see Additional file 1]. An eight-point Likert-type scale (1 = most willingly; 8 = most unwillingly) was used to rate willingness to share a social space. Social distance was calculated as the sum of the seven items (i.e., a possible score of 8–56). Given the arbitrary nature of the measure, it was rescaled to lie between 0 (low stigma) and 100 (high stigma) presenting each participants score as a percentage of the scale maximum (%SM).

#### Survey

The survey was divided into three sections. The first section collected demographic details. The second section presented a series of three random vignettes, each portraying one of the 15 possible descriptions of the hypothetical person. Following each vignette was the adapted social distance scale. The final section was a measure of HIV/AIDS knowledge.

The repeated measurement in this instance, however, was only used as a device to increase the sample size, (to overcome the relatively small number of students in the second year of the medical course). There was an implicit trade-off between increasing the number of judgments made about the hypothetical person and the potential redundancy associated with repeated measurement. In order to prevent a set response to each version of the hypothetical person, surveys were individually printed and constructed so that each complete survey elicited responses to three versions of the hypothetical person: one with AIDS, one with leukaemia, and one with no disease. Furthermore, no co-characteristic occurred in any survey more than once. It also meant that in any block of 360 surveys, the presentation was counter-balanced such that no two participants would read descriptions of the same three versions of the hypothetical persons. The 360 possible three-vignette combinations were shuffled using a random number generator prior to distribution.

The adaptation and (back) translation of the vignettes went through a series of iterations and the final survey was field tested for readability and sense with 15 medical students.

### Procedure

In a single morning, at the conclusion of lectures, research assistants were provided with an opportunity to address concurrent classes of second year medical students, and invite them to participate in a survey on student attitudes. A plain language statement of the research was distributed and it was explained both orally and in the plain language statement that participation was voluntary and the responses were entirely anonymous. Students who did not wish to participate were invited to leave the lecture theatre. However, mindful of the potential for coerced participation through such a public form of non-participation, students were also given alternative forms of non-participation. Specifically, they were informed that they could remain and choose not to submit the survey form or submit a blank form. Separate signed consent was not sought, because it was regarded as culturally inappropriate and would raise unnecessary concerns about anonymity.

Research assistants distributed the survey forms serially from a randomly ordered master pile. Students were given 15 minutes to complete the surveys, and encouraged not to look at others' responses. The distribution of vignettes across the 15 possible disease and co-characteristic combinations about 'A' can be found in Additional file 2.

### Data Analysis

All analyses were conducted using of Intercooled Stata version 8.2. In the first instance the scale properties of the social distance scale were examined, and then the bivariate relationship between the independent factors (disease characteristic and co-characteristic), the covariates, and social distance. The effect of the order of the presentation of a vignette on social distance was included as a possible confounding variable. Although the presentation of vignettes was randomised, participants would have been unfamiliar with these particular descriptions, and it was felt that repeated exposure to the task may itself influence the judgements.

A multivariable model in which the effects of the independent factors (and interaction effects) were estimated, controlling only for those covariates that were shown to have statistically significant effects on stigma in the bivariate tests. Adjusted cell means (without the model constant) were calculated and the effect of the layering of stigma examined.

The multivariable modelling was, conceptually, a straightforward analysis of social distance as a function of (a) the disease characteristic and (b) the co-characteristic. It was slightly complicated by the repeated measurement of social distance within participants – each of whom was asked to respond to three separate vignettes. The clustering of judgements associated with the repeated measurement of social distance within participants was handled using a two-level, maximum likelihood, multilevel regression analysis. In the model, social distance associated with judgements were represented at level one, and participants were represented at level two [38,39].

### Ethics

The study was approved by the Deakin University Human Research Ethics Committee.

## Results

### Bivariate and multivariable analyses

The internal reliability of the social distance scale was confirmed using Cronbach's alpha (α =.91) [see Additional file 3]

The social distance scale was linearly transformed to lie in the interval 0–100 (0 representing minimum social distance, 100 representing maximum social distance). Social distance scores occurred over the entire range of the scale, with a mean social distance of 30.4.

Bivariate analyses were conducted to examine the relationship between social distance and each of the independent variables and covariates separately, including the order of presentation (Table 1). In virtue of the clustered nature of the data, the bivariate analyses were performed using a maximum likelihood, multilevel (variance components) regression analysis (Table 1).

**Table 1:** Bivariate analysis of social distance, adjusting for the effect of repeated judgements within participants^1^

Variable	n P/J	Unstandardised Coefficient	Standard Error	p	95% CI
Order (base = First)	352/1044				
Second		4.81	1.7	.005	1.47 – 8.15
Third		6.06	1.7	.000	2.73 – 9.39
Age	346/1026	0.00	1.05	.997	-2.06 – 2.06
Sex (base = Male)	339/1005				
Female		-.78	1.81	.666	-4.34–2.77
Knowledge	352/1044	-.25	.50	.615	-1.24 – 0.73
Disease (base = None)	352/1044				
Leukaemia		-6.99	1.63	.000	-10.18 – -3.80
AIDS		7.39	1.63	.000	4.20 – 10.58
Co-Characteristic (base = None)	352/1044				
BT		.07	1.96	.972	-3.78 – 3.92
CBD		2.34	1.96	.235	-1.52 – 6.19
CS		19.06	1.98	.000	15.17 – 22.94
IDU		25.13	1.96	.000	21.29 – 28.97

^1^The analysis examined the order of the vignette presentation (Order); participants' age, sex, and knowledge of HIV/AIDS, the disease in the vignette, and the presence of a co-characteristic: none, blood transfusion, commercial blood donation (CBD), commercial sex (CS), and injecting drug use (IDU). The sample size for each analysis is also shown: both the total number of participants (P) and the total number of social distance judgments (J).

The analysis showed the order of presentation to have a statistically significant effect on participants' judgments about social distance. As a percentage of the scale maximum, vignette two was, on average, 4.8% higher on the social distance than vignette one (p = .005), and vignette three was 6% higher (p < .001). That is, social distance increased significantly with the increasing order of presentation. None of the covariates (age, sex or HIV/AIDS knowledge) had a statistically significant effect on social distance.

The results were very different for the independent factors, Disease and Co-Characteristic. For the Disease factor, Leukaemia was significantly less stigmatising than no disease, resulting in an average reduction in social distance of 7% (p < .001). In contrast, AIDS was significantly more stigmatising than no disease resulting in an average increase in social distance of 7% (p < .001). As anticipated, having a blood transfusion did not significantly increase social distance above the base category. Likewise, commercial blood donation did not significantly increase social distance (p = .235). In contrast, having engaged in a commercial sex transaction increased social distance by an average of 19% of the scale maximum (p < .001), and having engaged in IDU increased social distance by an average of 25% (p < .001).

In the multivariable model of social distance the order of presentation was controlled for. Participants' sex, age, and knowledge of HIV/AIDS, however, were not included as covariates in virtue of their small and statistically non-significant association with social distance in the bivariate models. The focus of the multivariable model was the factors of Disease and Co-characteristic and their interaction effects. Table 2 shows the results of the maximum likelihood, multilevel (variance component) regression analysis, which takes account of the clustering effect of the repeated measures within participants. The intra-class correlation was .35 indicating that a substantial proportion of the variation in social distance was attributable to within participant effects.

**Table 2:** Multivariable analysis of social distance, adjusting for the effect of repeated judgements within participants and controlling for the order of vignette presentation

Variable	Unstandardised Coefficient	Standard Error	p	95% CI
Order (base= First)				
Second	4.91	1.30	.000	2.37–7.44
Third	5.73	1.29	.000	3.20–8.26
Disease (base = None)				
Leukaemia (L)	2.48	3.26	.447	-3.91 – 8.86
AIDS	24.32	3.31	.000	17.83 – 30.81
Co-Characteristic (base = None)				
BT	5.00	3.27	.126	-1.40 – 11.41
CBD	9.13	3.26	.005	2.74 – 15.51
CS	30.66	3.27	.000	24.34 – 37.06
IDU	43.84	3.25	.000	37.48 – 50.20
Interaction Effects				
L × BT	-1.55	4.72	.742	-10.81 – 7.71
L × BS	-6.77	4.72	.151	-16.01 – 2.47
L × CS	-10.35	4.77	.030	-19.69 – -1.01
L × IDU	-25.57	4.73	.000	-34.84 – -16.30
AIDS × BT	-13.17	4.73	.006	-22.44 – -3.89
AIDS × CBD	-13.15	4.78	.004	-22.51 – -3.79
AIDS × CS	-25.13	4.76	.000	-34.46 – -15.79
AIDS × IDU	-31.64	4.73	.000	-40.91 – -22.38

ICC = .35

In the multivariable analysis the two factors and the interaction were statistically significant. The number of tests (i.e. 14) that were conducted raises some issues about Type I errors in multiple comparisons. For reasons discussed by Cook and Farewell (1996), however, this was not regarded as problematic. The core questions revolved around Leukaemia, AIDS, IDU, CS, and their interactions. Most of the effects appeared to fall clearly one way or another. Even using as conservative a correction for multiple comparisons as the Bonferroni adjustment, the only questionable result would be the interaction effect between Leukaemia and CS – for which there was not, in any case, a specific *a priori *hypothesis.

In keeping with the literature, however, the pattern of results suggested that AIDS, IDU, CS, and to a lesser degree CBD, were each highly stigmatising. They increased the social distance score between 9% and 44% of the scale maximum. In contrast, BT in isolation had no statistically significant effect to the level of stigma, confirming its suitability as a control co-characteristic. The pattern of interaction also showed a moderating effect, as anticipated by Reidpath and Chan (2005). Specifically, the stigma associated with the co-occurrence of two highly stigmatised attributes was not simply a summation of the stigma of each; rather, the stigma appeared to lie generally between the stigma of one and the stigma of the sum.

In contrast to the bivariate analyses, leukaemia in isolation did not have a statistically significant moderating effect on levels of stigma, however, it had a statistically significant moderating effect on the stigma of IDU, but possibly not on the stigma of CS. In addition, commercial blood donation as a co-characteristic decreased the level of stigma associated with AIDS.

Following the approach described by Reidpath and Chan [26], the layering of the stigmas between disease and co-characteristics was examined. Using the figures presented in Table 3, cell means (%SM) were calculated as the sum of the disease coefficient, co-characteristic coefficient, and the interaction effect (see Table 3).

**Table 3:** Model estimates (MLE) of social distance scores as a function of co-characteristics and disease conditions.

	Disease Conditions		
	
Co-Characteristics	No Disease	Leukaemia	AIDS
None	0.00	2.48	24.32
BT	5.00	5.93	16.17
CBD	9.13	4.83	19.72
CS	30.66	22.78	29.85
IDU	43.84	20.75	36.52

Very different arrangements of layering were observed between AIDS and the various co-characteristics, each of which are discussed in turn. It should be noted, however, that the layering effects are based on estimates with often wide confidence intervals and this limits the extent to which one can interpret the results.

### Layering results

#### AIDS and IDU

**Figure 2:**
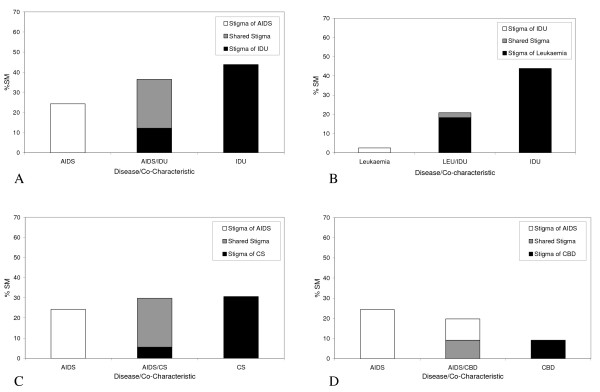
**The layering arrangements between the stigmas of diseases and co-characteristics**. A. The layering of AIDS and injecting drug use (IDU). B. The layering of leukaemia and injecting drug use (IDU). C. The layering of AIDS and commercial sex (CS). D. The layering of AIDS and commercial blood donation (CBD).

Figure 2a shows the arrangement of stigmatisation of the person with (1) AIDS in the absence of a co-characteristic (left hand bar), (2) IDU in the absence of a disease (right hand bar) and (3) the combination of AIDS and IDU (middle bar). The height of the bars represents the effect of these diseases/co-characteristics on social distance. AIDS has the lowest effect on social distance, IDU has the greatest effect on social distance, and the combination of the two factors (i.e. AIDS and IDU) sits between the two.

Chi-square test confirmed that the difference on stigma levels between AIDS alone and the combination of the two factors is statistically significant, χ^2^(1) = 14.02, p < .001. The difference on stigma levels between IDU alone and the combination of the two factors is statistically significant (χ^2^(1) = 5.22, p = .022). This layering arrangement is also consistent with leukaemia and IDU (see Figure 2b), where the level of stigma of the combination of IDU and leukaemia is less than half of the stigma attached to IDU alone, χ^2^(1) = 50.61, p < .001.

Figure 2a shows the social distance score shown in the middle bar as a composite of the social distance associated with IDU alone, and the 'shared' social distance of IDU and AIDS. The smaller portion of the total social distance shown in the middle bar is uniquely associated with IDU (33%). The greater portion of the social distance (67%) is 'shared' and associated with the interaction between IDU and AIDS.

There is no stigma that is uniquely associated with AIDS. It is by reference to Table 2 that one possible explanation for this can be found. If there were no statistically significant interaction effects between AIDS and IDU, then the social distance associated with a person having both characteristics would be simply additive (i.e. the AIDS coefficient, 24.32, plus the IDU coefficient, 43.84, equals 68). However, there is a statistically significant negative interaction effect (the coefficient IDU/AIDS), indicating that the social distance associated with AIDS and IDU must be less than the sum of the two unique social distance scores. Because the negative effect of the interaction is greater than the social distance associated with AIDS but less than the social distance of IDU, any unique AIDS social distance disappears entirely, and appears as shared between IDU and AIDS.

#### AIDS and CS

Figure 2c presents the arrangement of stigmatisation of the person with (1) AIDS in the absence of a co-characteristic (left hand bar), (2) CS in the absence of a disease (right hand bar), and (3) the combination of AIDS and CS (middle bar). The heights of the three bars suggest that AIDS has the lowest effect on social distance while the effect of CS and the combination of the two factors have similar effects on social distance. Chi-square test confirms that AIDS has a significantly lower effect on social distance than CS (χ^2^(1) = 3.93, p = .047), but the difference in stigma levels is not statistically significant between AIDS and the two factors combined (χ^2^(1) = 2.84, p = .092). Similarly, there are no statistically significant difference in the effect on social distance between CS and the two factors combined (χ^2^(1) = .06, p = .804). This is in sharp contrast with the effects observed in the control disease where the stigma of CS is significantly higher than the stigma of CS and leukaemia combined (χ^2^(1) = 5.59, p = 0.018).

Like the layering arrangement of IDU and AIDS, the middle bar in Figure 2c is mostly made up of the 'shared' stigma of CS and AIDS and a small level of stigma that is uniquely associated with CS (19%). No stigma that is uniquely associated with AIDS is observed. The negative interaction effect between CS and AIDS (see Table 2) may again hold the key to an explanation that will be discussed later.

#### AIDS and CBD

Figure 2d shows the arrangement of stigmatisation of the person with (1) AIDS in the absence of a co-characteristic (left hand bar), (2) CBD in the absence of a disease (right hand bar) and (3) the combination of AIDS and CBD (middle bar). In contrast to the previous arrangements, AIDS has the highest effect on social distance while CBD has the lowest effect on social distance; the effect of the combination of the two factors sits in the middle. This arrangement is the complete reversal of that observed between AIDS and IDU.

Unlike the layering arrangements already discussed, the stigma associated with AIDS alone made up 54% of the total stigma in the middle bar, while the 'shared' stigma of the co-characteristic and AIDS makes up 46% of the stigma. There is no stigma that is uniquely associated with CBD. This layering is similar to that of AIDS and the controlled co-characteristic of blood transfusion, where AIDS makes up 69% of the stigma and Blood transfusion 31%.

### Additional post-hoc analyses

Previous studies have commonly reported that the stigma attached to people with AIDS and a co-characteristic is significantly more stigmatising than the control disease with the same co-characteristic. Additional chi-square analyses confirmed that this is true for the current dataset. As such, AIDS layered with all four co-characteristics were significantly more stigmatising than leukaemia layered with the same co-characteristics. The results by co-characteristics are as follows: IDU (χ^2^(1) = 23.62, p < .001), CS (χ^2^(1) = 4.51, p = .033), CBD (χ^2^(1) = 20.78, p < .001), and BT (χ^2^(1) = 9.91, p = .002). Chi-square results also shows that AIDS with BT was significantly more stigmatising than BT alone (χ^2^(1) = 7.58, p = .006).

## Discussion

The observed inter-relationship between HIV/AIDS stigma and the three co-stigmas was as hypothesised, and conforms to the last of the three models proposed[26]. The stigma of HIV/AIDS and the three co-stigmas are neither strictly additive nor completely overlapping. Instead, the layering arrangement showed a portion of stigma that is a 'shared' effect of the disease stigma and the co-stigma as well as independent effects.

An important finding is that people with AIDS are not the ones who are most stigmatised. The person who is an injecting drug user without an accompanying disease is most stigmatised. A drug user with AIDS ranks second, followed by CS with no disease, and then CS with AIDS. This is a finding commonly missed by research with the traditional focus which attends to HIV/AIDS as the most critical source of stigma (e.g. [17,21,40]. The results highlight the need to understand the stigma of HIV/AIDS in a wider context than the disease itself.

The magnitude of the 'shared' effect relative to the independent effects of a disease or co-characteristic suggests the extent to which the meanings of the two stigmas overlap, or may be derived from one another. For a number of reasons, one might suspect that the disease stigma of HIV/AIDS is a derivative of the negative meanings attached to IDU and CS, and not the other way around. One can see for instance that in the AIDS/CS and AIDS/IDU interaction that there is a complete absence of a 'unique' AIDS stigma. The contribution of AIDS to the overall stigma is entirely embedded within the interaction. This explanation is consistent with the natural history of the epidemic in China where it is still a predominately IDU epidemic (particularly in places like Guangzhou) [27,41]; and the stigmatisation of IDU and CS predates the stigmatisation of HIV/AIDS [42-44]. It is highly likely that for most injecting drug users infected with HIV, the drug habit preceded infection, not the other way around [40,45]. This logically places CS and IDU first in the causal order, followed by AIDS.

Another challenge to the orthodox understanding of HIV/AIDS stigma is the statistically significant drop in the levels of stigma between an injecting drug user with no disease and an injecting drug user with AIDS (see Figure 2a). One could interpret the results as showing that having AIDS actually reduces the overall social distance of an injecting drug user. The analogous relationship between IDU and the control disease (leukaemia) suggests that this may be a general (perhaps sympathetic) reaction (Figure 2b). This merits further investigations as does the contrary finding that the moderating effect of illness on stigma is missing in the layering of AIDS and CS.

The results have important implications for the delivery of health care in settings where the co-characteristics are more stigmatising than AIDS, and the co-characteristics are the strongest determinants of infection. In these settings most PLWHA attending a health service will be positive in virtue of being a drug user or having engaged in CS. If stigma is a barrier to treatment and care, it is likely that reducing the stigma associated with AIDS alone is not enough if in the minds of health carers the occurrence of AIDS marks a person as having the even more stigmatised co-characteristic of CS or IDU. Indeed, focussing on the AIDS alone may backfire and reduce any sympathetic reaction that occurs towards an HIV-positive drug user.

That AIDS in the absence of an 'innocent' explanation such as CBD is more stigmatising is also important (see Table 3). It suggests that an 'innocent' explanation for being sero-positive pre-empts the possibility that a person had acquired AIDS as a by-product of some other stigmatising behaviour. Conversely, having AIDS without any details about the mode of transmission leaves people speculating about the mode of transmission. A 'spill-over' effect seems to occur from the un-stated but suspected stigma of a negative co-characteristic; a further indication that the stigma of co-characteristics such as IDU and CS are embedded within the being HIV-positive. It also has interesting implications for whether a PLWHA would or should reveal the source of infection.

Speculatively, the data also speak to the constructs of 'guilty' and 'innocent' victims of AIDS described in the literature [46-48]. If the dichotomy is absolute, the stigma for 'innocent' PLWHA should not significantly exceed that of (1) the 'innocent' leukaemia victim with the same co-characteristic, or (2) the 'innocent' person with only the co-characteristic. Yet, such differences were found in the data, indicating that there may be a limit to the stigma exemption for 'innocent' victims of AIDS. However, the finding may be due to the study design itself. The disease and co-characteristic factors presented in each version of the vignette were not mutually exclusive. Therefore, participants could not be certain that a HIV-positive person who engaged in blood transfusion did not also engage in other unspecified stigmatised behaviours. This, of course approximates the ambiguity of real life circumstances where information about transmission is often unavailable even to health care professionals. Instead of the dichotomised construct of guilty versus innocent, one interpretation of the overall findings is that there might be a gradient in the levels of guilt attached to different categories of people with AIDS; with those who engage in IDU being the most guilty and those who could prove their 'innocence' being the least guilty.

### Limitations

The research method used in the current study provides a promising way for understanding the complexity of HIV/AIDS-related stigma in a health care setting. The results are not, however without limitations. The vignettes describe a person who was in all respects culturally perfect except for the minor 'lapses' of drug use and the occasional visit to a sex worker. This description is probably a typical of the underlying population it tries to represent and the results must therefore be considered in this light. The participants were also a cohort of medical students from only one university, raising questions of generalisability. Both issues are, however, in many ways secondary. Developing and testing an analytic framework for understanding the interrelationships between HIV/AIDS stigma and a selected number of its co-stigmas was more important and limited generalisability of the actual results are in this case of less concern. Nonetheless, more testing is required to examine the adaptability of the approach to other settings. In other settings, of course, the dynamics of layering may vary (see for example, [49,50]. The diversity of layering would only serve to highlight the complexity of issue of stigma and the need for developing more context specific understandings of the issue.

Another limitation is the operational definition of 'stigma' in terms of social distance. Although social distance is perhaps one of the most empirically valid means for measuring stigma, further studies should look at incorporating measures that tap into other aspects of stigma when examining the issue of layering. The approach described here could be adapted for use with other attitudinal scales that measure other aspect of stigma.

The current study also restricted the co-characteristics that were explored. Other factors that merit investigation include the co-stigma of homosexuality, the gender of the person portrayed in the vignette, and the stigma associated with sex workers as opposed to their clients. The modelling of triple stigmas (e.g. IDU, CS and AIDS) could also be a fruitful line of future inquiry. As with all attitudinal studies, cautions should be made when inferring behaviour from the findings.

A final and important issue is with the variability in the data themselves, shown by the wide confidence intervals associated with the parameter estimates. Under these circumstances, particular inferences about the nature of the layering should be regarded as broadly speculative, and indicative of potentially useful, future lines of inquiry.

Notwithstanding these limitations, the study presents a potentially useful way of considering the layering of stigmatised characteristics.

## Conclusion

The findings of this study indicate that, far from being a coherent singular entity, HIV/AIDS related stigma overlaps with a number of co-stigmas associated with the modes of disease transmission. Variations in the ways different co-stigmas overlapped with HIV/AIDS stigma were examined, with each having different implications for stigma interventions. The most important was the extent to which the co-stigmas of IDU and CS were layered upon the disease stigma of HIV/AIDS suggesting that the co-stigmas should be taken into account in the development and implementation of HIV/AIDS stigma interventions. Although more work is needed, the research methods used in the current study provided a way that would hopefully contribute to a better means of analysing the nature of stigma as it relates to different HIV/AIDS epidemics.

## Abbreviations

Acquired Immune Deficiency Syndrome (AIDS)

Blood Transfusion (BT)

Commercial blood donation (CBD)

Commercial sex (CS)

Human immunodeficiency virus (HIV)

Injecting drug use (IDU)

People living with HIV/AIDS (PLWHA)

## Competing interests

The author(s) declare that they have no competing interests.

## Authors' contributions

DR contributed to the initial conception of the study, contributed to the study design and statistical analysis. KYC conceived of the study and was principally responsible for the study design, statistical analysis and the drafting of the manuscript. KLZ and YY worked on the cultural adaptability of the survey and data collection. All authors have provided feedbacks on the drafts and approved the final manuscript.

## Pre-publication history

The pre-publication history for this paper can be accessed here:



## Supplementary Material

Additional file 1 Scale Items. Click here for file

Additional file 2 The distribution of vignettes across the 15 possible disease and co-characteristic combinations about person 'A'. Click here for file

Additional file 3 Reliability of the adapted social distance scale based on results of the first vignette read by participants. Click here for file
