# Supplementary material for: Disentangling the stigma of HIV/AIDS from the stigmas of drugs use, commercial sex and commercial blood donation – a factorial survey of medical students in China
Source: BMC Public Health. 2007 Oct 5;7:280. doi: 10.1186/1471-2458-7-280 (PMC2180176; doi:10.1186/1471-2458-7-280)
Supplement: Additional file 1 — Scale Items. [file 1471-2458-7-280-S1.doc]

The social distance scale adapted from Kelly and colleagues:

1. If you met ‘A’, would you be willing to strike up a conversation with him?
2. Would you attend a party where ‘A’ was present?
3. Which you attend a party where ‘A’ was preparing food?
4. Would you be willing to work in the same office with ‘A’?
5. If you were a friend of ‘A’s, would you be willing to continue to friendship at this time?
6. ‘A’s lease is up in two months. If you were his landlord, would you renew his lease?
7. Would you allow your children to visit ‘A’ in his home?

Questions on knowledge of HIV/AIDS Transmission:

1. HIV/AIDS is curable?
2. You can get HIV from kissing a person who is infected with the virus?
3. You cannot get HIV from touching a person who is infected with the virus?
4. You can get HIV from a needle stick injury containing blood infected with the virus?
5. You can get HIV from oral-faecal contamination?
6. You can get HIV from unprotected sex?
7. You can get HIV from a mosquito bite?
8. Using a condom during sexual intercourse provides protection against HIV?
9. You can tell if a person has HIV by the way they look?
10. An infant can get HIV breast-feeding from a mother who has HIV?
11. You cannot get HIV from an HIV positive person who is on anti-retroviral?
