# Supplementary material for: Disentangling the stigma of HIV/AIDS from the stigmas of drugs use, commercial sex and commercial blood donation – a factorial survey of medical students in China
Source: BMC Public Health. 2007 Oct 5;7:280. doi: 10.1186/1471-2458-7-280 (PMC2180176; doi:10.1186/1471-2458-7-280)
Supplement: Additional file 2 — The distribution of vignettes across the 15 possible disease and co-characteristic combinations about person 'A'. [file 1471-2458-7-280-S2.doc]

|  | Disease Characteristic | | | |
| --- | --- | --- | --- | --- |
| Co-Characteristic | Nil | Leukaemia | AIDS | **Row Total** |
| Nil | 69 | 73 | 68 | **210** |
| Blood Transfusion (BT) | 70 | 72 | 69 | **211** |
| Commercial blood donation (CBD) | 71 | 71 | 70 | **212** |
| Commercial Sex (CS) | 70 | 66 | 72 | **208** |
| Injecting Drug User (IDU) | 72 | 70 | 73 | **215** |
| **Column Total** | **352** | **352** | **352** | **1056** |
