# Supplementary material for: Disentangling the stigma of HIV/AIDS from the stigmas of drugs use, commercial sex and commercial blood donation – a factorial survey of medical students in China
Source: BMC Public Health. 2007 Oct 5;7:280. doi: 10.1186/1471-2458-7-280 (PMC2180176; doi:10.1186/1471-2458-7-280)
Supplement: Additional file 3 — Reliability of the adapted social distance scale based on results of the first vignette read by participants. [file 1471-2458-7-280-S3.doc]

|  | Reliability Characteristics | | |
| --- | --- | --- | --- |
| Scale Item | Item-test correlation | Item-rest correlation | α if item is deleted |
| 1. If you met ‘A’, would you be willing to strike up a conversation with him? | .77 | .69 | .9 |
| 2. Would you attend a party where ‘A’ was present? | .82 | .76 | .89 |
| 3. Which you attend a party where ‘A’ was preparing food? | .84 | .76 | .89 |
| 4. Would you be willing to work in the same office with ‘A’? | .87 | .80 | .88 |
| 5. If you were a friend of ‘A’’s, would you be willing to continue the friendship at this time? | .78 | .71 | .90 |
| 6. ‘A’'s lease is up in two months. If you were his landlord, would you renew his lease? | .77 | .67 | .90 |
| 7. If you had children, would you allow your children to visit ‘A’ in his home? | .82 | .73 | .89 |
